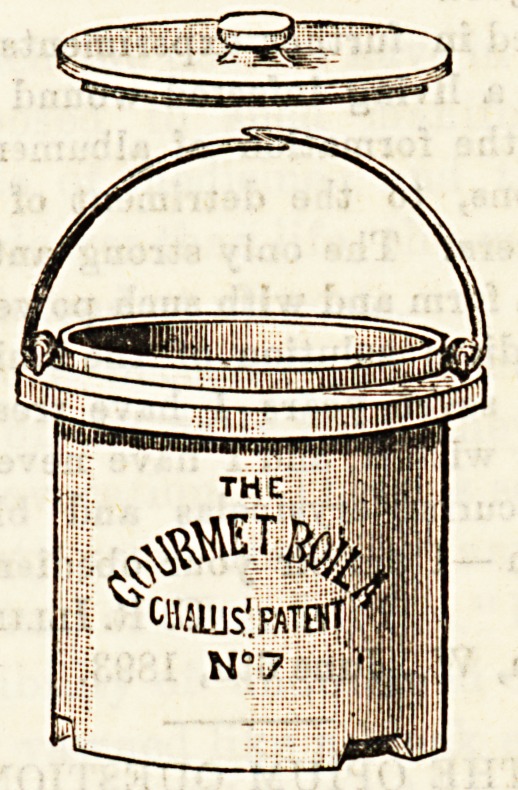# The "Gourmet" Boiler

**Published:** 1893-06-17

**Authors:** 


THE "GOURMET" BOILER.

An invention of a somewhat similar nature to the one described above is the "Gourmet" boiler. This little vessel will be found most useful in the preparation of beef tea. It is made of glazed earthenware, and can be had in various sizes to fit any saucepan, the price ranging from 1s. to 4s. The saucepan is half-filled with water, and the food to be cooked placed in the boiler. A uniform temperature can thus be obtained, and the vessel may be left on the fire all night with no possibility of porridge, soup, or beef tea becoming burnt or in any way spoilt. For preparing food for children and invalids we most strongly recommend its use, and feel sure that one trial will ensure its adoption. The inventors are Messrs. Nye and Co., 139, Oxford Street, by whose kind permission we are able to give a drawing of this useful little pot.

## Figures and Tables

**Figure f1:**